# Supplementary material for: NOTO Transcription Factor Directs Human Induced Pluripotent Stem Cell-Derived Mesendoderm Progenitors to a Notochordal Fate
Source: Cells. 2020 Feb 24;9(2):509. doi: 10.3390/cells9020509 (PMC7072849; doi:10.3390/cells9020509)
Supplement: Supplementary file 1 [file cells-09-00509-s001.pdf]

cells

Supplementary Materials

NOTO transcription factor directs human induced pluripotent stem cell-derived mesendoderm progenitors to a notochordal fate

Pauline Colombier [1], Boris Halgand [1,2] Claire Chédeville [1], Caroline Chariau [3], Valentin François-Campion [4], Stéphanie Kilens [4], Nicolas Vedrenne [1], Johann Clouet [1,5], Laurent David [3,4,†], Jérôme Guicheux[1,2,†] and Anne Camus [1,*,†]

1 INSERM UMR 1229, RMeS, Université de Nantes, ONIRIS, Nantes, F-44042, France
2 CHU Nantes, PHU 4 OTONN, Nantes, F-44042, France
3 Nantes Université, CHU Nantes, INSERM, CNRS, SFR Santé, FED 4203, Inserm UMS 016, CNRS UMS 3556, Nantes, F-44042, France
4 Nantes Université, CHU Nantes, INSERM, CRTI, UMR 1064, ITUN, Nantes, F-44042, France
5 CHU Nantes, Pharmacie Centrale, PHU 11, Nantes, F-44042, France
* Correspondence: Anne.Camus@univ-nantes.fr; Tel.: + 33 02 40 41 29 43
† Co-senior author

Received: 03 January 2020; Accepted: 19 February 2020; Published: date

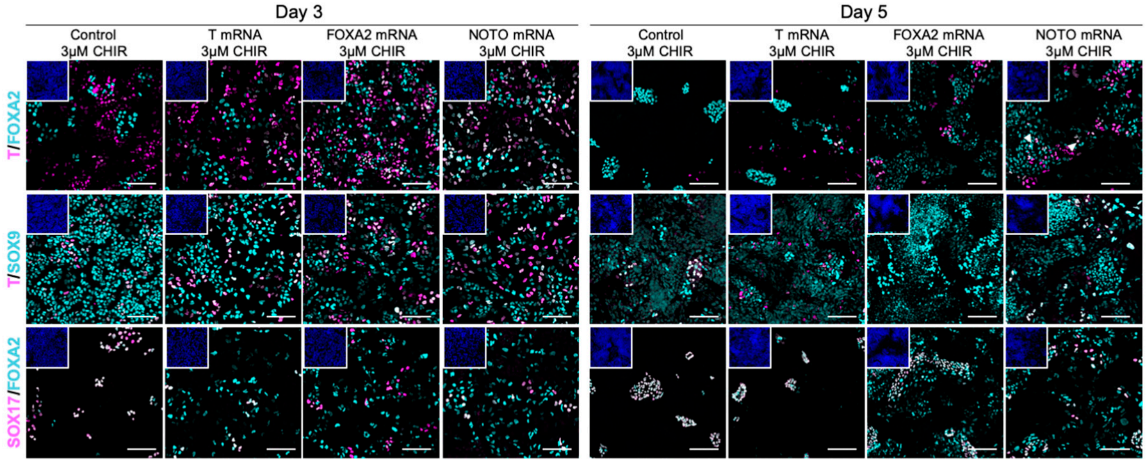

Figure S1. Differentiation of mesendoderm progenitors following *T*, *FOXA2* and *NOTO* mRNA transfections. Immunostainings of T+/FOXA2+, T+/SOX9+ and SOX17+/FOXA2+ positive cells at day 3 and day 5 in *T*-, *FOXA2*- , and *NOTO*- transfected conditions. Insets are showing nuclei stained with Hoechst. Scale bars: 100 µm.

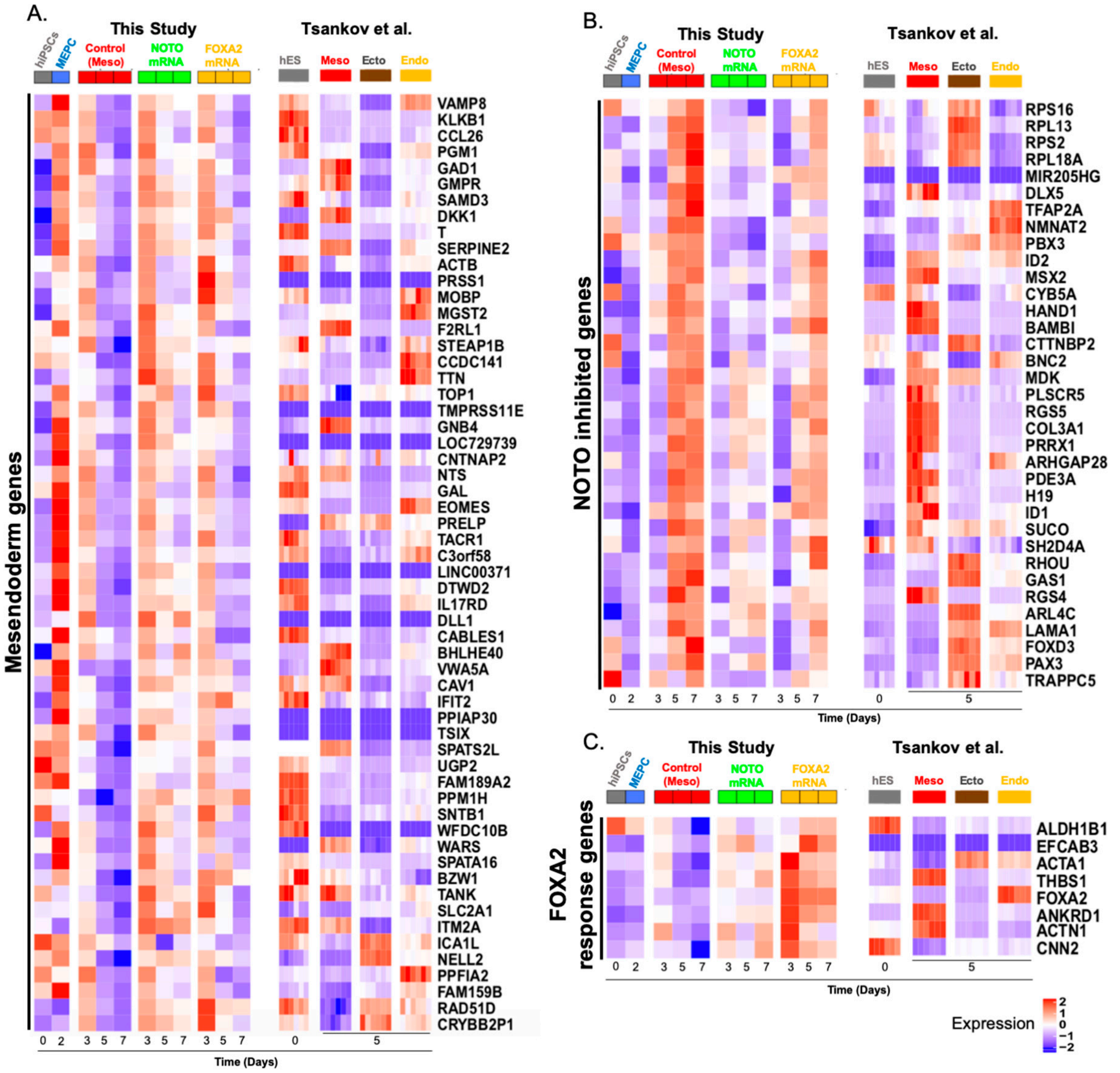

Figure S2. Details of the three remaining transcriptomic clusters presented in figure 8C. (**A**) Expression levels of mesendoderm genes during the course of *NOTO*- and *FOXA2*-driven MEPC differentiation (this study) and in hESC-derived mesoderm, ectoderm and endoderm [51] ; (**B**) Expression levels of NOTO inhibited genes during the course of *NOTO*- and *FOXA2*-driven MEPC differentiation (this study) and in hESC-derived mesoderm, ectoderm and endoderm [51]; (**C**) Expression levels of FOXA2 response genes during the course of *NOTO*- and *FOXA2*-driven MEPC differentiation (this study) and in hESC-derived mesoderm, ectoderm and endoderm [51]. Expression levels are presented as a gene-centered heatmap with lower values as blue, median as white and higher values as red colors.

Table S1. List of reagents used for hiPSCs culture and differentiation.

Reagents	Reference	Manufacturer
**TryplE**	12605-1010	Life technologies
**Rock inhibitor**	1254	Tocris
**CHIR99021**	100-1386	Axon MedTech
**Activin A**	130-097-611	Miltenyi Biotec
**lipofectamine RNAiMAX**	13778-150	Life technologies
**FGF2**	100-18B	Peprotech
**SHH**	130-095-721	Miltenyi Biotec

Table S2: List of Taqman Assays and Primer sequences for RT-qPCR analysis by SYBR GREEN technology, relative to Figure 2, 3, 4, 5, 6, and 7. Forward (Fp) and reverse (Rp) primers have been designed in the 3'UTR region in order to distinguish *T*, *FOXA2* and *NOTO* endogenous transcripts of the synthetic mRNAs.

Gene Name	Taqman Assay
BRACHYURY / T	Hs00610080-m1
CA12	Hs01080909-m1
CDH2	Hs00983056_m1
CER1	Hs 00193796-m1
EOMES	Hs00172872_m1
FN1	Hs01549976_m1
FOXA1	Hs04187555_m1
FOXA2	Hs00232764_m1
FOXF1	Hs00230962-m1
FOXJ1	Hs00230964_m1
GAPDH	Hs99999905_m1
GLI1	Hs00171790_m1
GSC	Hs00418279_m1
KRT18	Hs02827483_g1
LEF1	Hs 01547250-m1
LEFTY1	Hs 00764128-s1
LGALS3	Hs00173587_m1
MILX1	Hs00430824_g1
NANOG	Hs04260366_g1
NODAL	Hs00415443_m1
NOGGIN	Hs00271352_s1
NOTO	Hs01377437_m1
POU5F1	Hs00999632_g1
SHH	Hs00179843_m1
SOX17	Hs00751752_s1
SOX2	Hs01053049_s1
SPRY1	Hs01391580_m1
TBX6	Hs00365539_m1

Gene Name		Nucleic sequence
BRACHYURY / T (3'UTR)	Fp-	ACATCGTGGACAGCCAGTA
	Rp-	GGAAGTTACTGAGGCTGCATTT
FOXA2 (3'UTR)	Fp-	CATGCCTGGCAGCTTGG
	Rp-	CTCTCTCACTTGTCCTCGATCC
GAPDH	Fp-	GCACCGTCAAGGCTGAGAAC
	Rp-	GGATCTCGCTCCTGGAAGATG
NOTO (3'UTR)	Fp-	CTGAGGGCAGCAGTTACAT
	Rp-	CTTCTGGTTGAGGAGGCTTT

Table S3. Antibodies and dilutions used for Immunofluorescence experiments

Primary Antibodies	References	Dilutions
Goat polyclonal anti-BRACHYURY / T	AF2085, R&D systems	0.5µg/ml
rabbit polyclonal anti-FOXA2	8186, Cell Signaling Technology	1/400
rabbit polyclonal anti-SOX9	AB5535, Millipore	1/1000
goat polyclonal anti-SOX17	AF1924, R&D systems	1/200
**Secondary Antibodies**	**References**	**Dilutions**
Alexa 488-conjugated donkey anti goat	A11055, Life technologies	1/1000
Alexa 594-conjugated donkey anti rabbit	A21207, Life technologies	1/1000

.
